# Repeated Intraperitoneal *α*-Radioimmunotherapy of Ovarian Cancer in Mice

**DOI:** 10.1155/2010/394913

**Published:** 2009-10-25

**Authors:** Jörgen Elgqvist, Håkan Andersson, Holger Jensen, Helena Kahu, Sture Lindegren, Elisabet Warnhammar, Ragnar Hultborn

**Affiliations:** ^1^Institute of Clinical Sciences, The Sahlgrenska Academy, University of Gothenburg, 413 45 Gothenburg, Sweden; ^2^PET and Cyclotron Unit, Rigshospitalet, Copenhagen, Denmark

## Abstract

The aim of this study was to investigate the therapeutic efficacy of *α*-radioimmunotherapy of ovarian cancer in mice using different fractionated treatment regimens. The study was performed using the monoclonal antibody MX35 F(ab′)_2_ labeled with the *α*-particle emitter ^211^At. *Methods*. Nude mice were intraperitoneally inoculated with ~1 × 10^7^ cells of the cell line NIH:OVCAR-3. Four weeks later 6 groups of animals were given 400 kBq ^211^At-MX35 F(ab′)_2_ as a single or as a repeated treatment of up to 6 times (*n* = 18 in each group). The fractionated treatments were given every seventh day. Control animals were treated with unlabeled MX35 F(ab′)_2_ (*n* = 12). Eight weeks posttreatment the animals were sacrificed and the presence of macro- and microscopic tumors and ascites was determined. *Results*. The tumor-free fractions (TFFs) of the animals, defined as the fraction of animals with no macro- and microtumors and no ascites, were 0.17, 0.11, 0.39, 0.44, 0.44, and 0.67 when treated with 400 kBq ^211^At-MX35 F(ab′)_2_ once or 2, 3, 4, 5, or 6 times, respectively. Repeated treatment 3 times or more resulted in a significantly higher (*P* < .05) TFF than compared to treatment once or twice. The presence of ascites decreased from 15 out of 18 animals in the group given only one treatment to zero for the 2 groups given 5 or 6 fractions. Treatment with unlabeled MX35 F(ab′)_2_ resulted in a TFF of zero. *Conclusion*. Weekly repeated intraperitoneal injections of tolerable amounts of activity of ^211^At-MX35 F(ab′)_2_ of up to 6 times produced increased therapeutic efficacy without observed toxicity, indicating a potential increase of the therapeutic index.

## 1. Introduction

Ovarian cancer frequently recurs on the peritoneal surface from remaining micrometastatic growth in spite of debulking surgery and systemic chemotherapy. External abdominal radiotherapy has proven unsuccessful due to absorbed dose limitations of normal tissues. Therefore, adjuvant locoregional treatment with intraperitoneal targeted ligands could be decisive in the treatment of remaining micrometastatic disease. Several studies have been performed on radioimmunotherapy (RIT) of ovarian cancer, mostly mAbs labeled with ^90^Y and ^131^I, in animals [1–6] and humans [7–12]. The *β*-emitting radionuclides however have too long a range for effectively treating microscopic tumors. Thus we believe it is important to continue our investigations of the efficacy of mAbs labeled with *α*-particle emitters when treating microscopic disease on the peritoneum [13]. In this study, as in a series of earlier studies [14–19], we used the *α*-particle emitter ^211^At, with a half-life of 7.21 hours, a mean range in tissue of ~62 *μ*m, and a mean linear energy transfer (LET) of ~111 keV/*μ*m. The half-life of this radionuclide makes it ideal for local treatment as the target cells are easily reached while the transfer of the radioimmunocomplex to the systemic circulation is delayed. The short range ensures a significant absorbed dose in microscopic tumors or even single cells. The high LET, together with the high relative biological effectiveness (RBE) of the *α*-particles necessitating only a few hits to devitalize the cell, indicates that only a small number of ^211^At-atoms have to be targeted to each cell [20, 21].

In this study we used the monoclonal antibody (mAb) MX35 F(ab′)_2_, which recognizes the sodium dependant phosphate transport protein 2b (NaPi2b) of ~90 kDa on ovarian cancer cells. We used an animal model mimicking the clinical situation with intraperitoneal RIT. The intraperitoneal approach allows a high absorbed dose to nonvascularized peritoneal tumor cells with low myelotoxicity as the clearance rate from the peritoneal cavity to the systemic circulation delays systemic exposure.

Fractionated external radiotherapy widens the therapeutic index compared to using a single fraction and higher absorbed doses can be delivered with acceptable toxicity. We hypothesize that this could be true for internal *α*-RIT.

## 2. Materials and Methods

### 2.1. Radionuclide

^211^At was produced by the ^209^Bi(*α*, 2n)^211^At reaction in a cyclotron (Scanditronix MC32 at the PET and Cyclotron Unit, Rigshospitalet, Copenhagen, Denmark) by irradiating a ^209^Bi target with 28-MeV *α*-particles. The ^211^At was isolated using a dry-distillation procedure [22].

### 2.2. Monoclonal Antibodies

MX35 is a murine IgG1-class mAb, developed and characterized at the Memorial Sloan-Kettering Cancer Center (MSKCC), Ny, USA. MX35 is directed towards the sodium dependant phosphate transport protein 2b (NaPi2b) of ~90 kDa on OVCAR-3 cells [23] and is expressed strongly and homogeneously on ~90% of human epithelial ovarian cancers [24]. A batch of MX35 F(ab′)_2_, produced by Strategic BioSolutions (Newark, USA) for clinical use, was provided by MSKCC.

### 2.3. Antibody Labeling

MAbs were labeled with ^211^At using the intermediate labeling reagent m-MeATE (*N*-succinimidyl 3-(trimethylstannyl)benzoate) [25]. Briefly, to a dry residue of ^211^At (50–100 MBq) was added a mixture of m-MeATE and N-iodosuccinimide in methanol: 1% acetic acid. This solution was then incubated for 20 minutes at room temperature and the labeling reaction was stopped by adding sodium ascorbate. The mAb MX35 F(ab′)_2_ was then added to the labeling mixture and conjugation was allowed to proceed for 20 minutes. Finally, the mAb fraction was isolated using a NAP-5 column (Amersham Biosciences, Uppsala, Sweden), resulting in a specific activity of 120 kBq/*μ*g, that is, 1 labeled mAb out of ~1200 mAbs.

### 2.4. Cell Line

The cell line OVCAR-3 (NIH:OVCAR-3, ATCC, USA) was used [26]. The cell line was obtained from the American Type Culture Collection, Rockville, MD, USA. The cells were cultured in T-75 culture flasks at 37°C in a humidified atmosphere of 95% O_2_/5% CO_2_ with RPMI-1640 cell culture medium supplemented with 10% fetal calf serum, 1% L-glutamine, and 1% penicillin-streptomycin.

### 2.5. Immunoreactivity of Antibodies

After conjugation, the immunoreactivity of the mAbs was analyzed in vitro by determination of the immunoreactive fraction, representing conditions of infinite antigen excess, which was derived from a plot of the total applied radioactivity divided by cell-bound radioactivity as a function of the inverse of the cell concentration [27].

### 2.6. Animals

We used 120 female, nude Balb/c nu/nu mice (Charles River Laboratories International Inc., Wilmington, MA, USA) in this study. The animals were housed at 22°C and 50%–60% humidity with a light/dark cycle of 12 hours. They were given autoclaved standard pellets and water ad libitum. All the experiments were approved by the Ethics Committee of the University of Gothenburg.

### 2.7. In Vivo Procedures and Study Groups

At the age of 5 weeks all mice were intraperitoneally inoculated with ~1 × 10^7^ OVCAR-3 cells suspended in 0.2 mL saline. Four weeks after cell inoculation the animals were divided into 7 groups. The animals in groups 1–6 were intraperitoneally injected with 400 kBq ^211^At-MX35 F(ab′)_2_ in 1 mL saline as a single or as a weekly treatment of 2, 3, 4, 5, or 6 times, respectively (*n* = 18 in each group). As controls (group 7), animals were treated once with unlabeled MX35 F(ab′)_2_ (*n* = 12). All the animals were thereafter weighed weekly. Eight weeks after the last treatment occasion for each group the animals were sacrificed by cervical dislocation and dissected. The abdominal cavity was opened and the presence of ascites and macroscopic lesions was judged as “yes” or “no”. Peritoneal biopsies were taken from the upper left quadrant since tumor propagation is most frequently seen in this area. Suspected lesions were also biopsied. All biopsies were processed for light microscopy and judged as “yes” or “no.” Animals dissected and judged were blinded from knowledge of exposure conditions. Differences in TFF and weight between the different study groups were tested using a 2-sample test for equality of proportions.

## 3. Results

The radiochemical yields were 30%–40% and the radiochemical purity was over 95% as determined by methanol precipitation and gel-permeability chromatography. The immunoreactivity measurements of the ^211^At-MX35 F(ab′)_2_ and OVCAR-3 cells gave an immunoreactive fraction of 0.95.

The TFFs of the study groups, defined as the fraction of animals with no macro- and microtumors and no ascites, were 0.17, 0.11, 0.39, 0.44, 0.44, and 0.67 when treated with 400 kBq ^211^At-MX35 F(ab′)_2_ as a single or as a repeated treatment regimen of 2, 3, 4, 5, or 6 times, respectively (Table 1). Repeated treatment of 3, 4, 5, or 6 times resulted in a significantly higher (*P *< .05) TFF than that compared to 1 or 2 treatment. The presence of ascites decreased from 15 out of 18 animals in the group given only one treatment to zero for the 2 groups given 5 or 6 repeated treatments. The presence of tumors did not decrease as drastically as the presence of ascites when the number of treatments increased. Treatment with unlabeled MX35 F(ab′)_2_ resulted in a TFF of zero.

The findings on the peritoneal biopsies at the time of dissection revealed both larger tumor cell clusters of several millimetres in diameter as well as clusters consisting of only a few tumor cells. The tumor cells were sometimes only loosely adhered to the peritoneum but had sometimes penetrated under the mesothelial cell layer.

The general condition of the animals seemed to be unaffected by the different treatment regimens, although the weights of the control animals were significantly higher (*P* < .05) than those of the animals given different regimens of ^211^At-MX35 F(ab′)_2_ (groups 1–6), due to the ascites production. No mutually significant difference (*P* > 0.5) in weight between the groups given different regimens of ^211^At-MX35 F(ab′)_2_ could be detected and no deaths occurred during followup.

## 4. Discussion

Fractionated radiotherapy in humans results in an increased therapeutic efficacy as compared to single doses and allows for increased total absorbed dose delivered to the target area. Mimicking such fractionation using RIT presents challenges with respect to physical half-lives and biodistribution. Reasons why fractionated RIT would appear promising are the possibility of reducing the systemic toxicity and hence increasing the maximum tolerated activity, achieving a more uniform absorbed dose distribution in the tumor, and increasing the therapeutic index. We have in a previous animal study found significantly less myelotoxicity dividing the injected activity into 3 fractions, with only a minor decrease in therapeutic efficacy [28]. In that study we also discussed the potential risk of treatment interruption in the human situation due to human antimouse antibody (HAMA) response. However, in our recently published phase I study in which we used a fragmented IgG1 mAb (MX35 F(ab′)_2_) we could not detect any signs of any HAMA response, indicating a low probability for an HAMA responses in potential future fractionated clinical RIT treatments [29]. In the present study we chose an activity well tolerated as a single injection (400 kBq), with a white blood cell recovery approximately within a week, to be repeated weekly for up to 6 times, that is, a total activity of up to 2400 kBq, not tolerated as a single injection [20]. An interval of 7 days was chosen from the bone marrow recovery data [28] as well as from logistics, that is, a weekly delivery of ^211^At. The rationale for choosing the fragmented mAb instead of the whole IgG in this study is due to 4 facts. (i) The fragmented mAb was the only clinical grade version of the mAb available at the time of the study; (ii) we have received an approval by the Swedish Medical Products Agency for carry through a phase I study with this fragmented mAb; (iii) we believe that the diffusion into tumors using the fragmented mAb is higher than compared to whole IgG; (iv) We believe that the immunogenicity of the fragmented mAb is lower than the whole IgG, reducing the risk for HAMA response, especially if repeated treatments are considered in the future.

In the series of experiments in this paper the efficacy expressed as the tumor-free fraction (TFF) was less than in previous studies from our group [14–19], but a significant total activity and TFF relation were shown without any signs of toxicity. The difference in the efficacy between the studies probably reflects varying proliferation of the injected cells resulting in different sizes of the tumor deposits at the time of treatment, that is, 4 weeks postinjection. Since the *α*-particle track length is limited to 60–70 *μ*m the size of the tumor cell clusters is crucial. In an earlier study [19] tumor dimensions were measured and the largest clusters at 4 weeks postinoculation were ~95 *μ*m, actually exceeding the *α*-particle path length. A significant peeling of the outermost cell layers of the tumor cell clusters and/or a uniform absorbed dose distribution does not seem probable since ~1/3 of the animals were not free of tumors in spite of up to 6 treatment fractions. In the interval of 400–1200 kBq in our earlier preclinical studies the TFF was not correlated with the administered activity. This could be explained by the saturation of the antigenic sites, which—according to the dynamic compartmental model introduced in one of those studies [17]—occurs within a few hours after the injection, resulting in a similar absorbed dose for those activity levels.

In our recently published phase I study on women in clinical complete remission after ovarian cancer occurrence we disclosed no marrow toxicity after an intraperitoneal injection of ~200 MBq ^211^At-MX35 F(ab′)_2_ in 1 L, which is in accordance with a low absorbed dose to the bone marrow derived from biokinetic data [29]. This, together with a low probability for HAMA response discussed above, could indicate a possibility of using a fractionated regimen in a phase II study now under planning.

In conclusion, weekly repeated intraperitoneal injections of tolerable amounts of activity of ^211^At-MX35 F(ab′)_2_ of up to 6 times produced increased efficacy without observed toxicity, indicating a potential increase of the therapeutic index.

## Figures and Tables

**Table 1:** Study groups and number of mice with macroscopic and microscopic tumors and ascites.

Group	*n*	Treatment	Number of treatments	Macroscopic tumors	Microscopic tumors	Ascites	TFF*
1	18	400^†^kBq^ 211^At-MX35 F(ab′)_2_ in PBS^‡^	1	11/18	15/18	15/18	0.17
2	18	400^†^kBq^ 211^At-MX35 F(ab′)_2_ in PBS^‡^	2	16/18	16/18	8/18	0.11
3	18	400^†^kBq^ 211^At-MX35 F(ab′)_2_ in PBS^‡^	3	11/18	11/18	5/18	0.39
4	18	400^†^kBq^ 211^At-MX35 F(ab′)_2_ in PBS^‡^	4	10/18	10/18	1/18	0.44
5	18	400^†^kBq^ 211^At-MX35 F(ab′)_2_ in PBS^‡^	5	10/18	10/18	0/18	0.44
6	18	400^†^kBq^ 211^At-MX35 F(ab′)_2_ in PBS^‡^	6	6/18	6/18	0/18	0.67
7	12	MX35 F(ab′)_2_ in PBS	1	12/12	12/12	10/12	0

*TFF : tumor-free fraction (i.e., fraction of animals with no macro- and microscopic tumors and no ascites). Injected activities were ^†^400 ± 14 kBq (mean ± SEM). ^‡^PBS : phosphate-buffered saline. The presence of macroscopic tumors and ascites was assesed by careful ocular inspection during dissection 2 mo after the last administration of the radioimmunocomplex. Microscopic tumor growth was assessed by conventional histopathology. Judgements were blinded from treatment information.
